# Autistic Traits and College Adjustment

**DOI:** 10.1007/s10803-022-05632-w

**Published:** 2022-07-07

**Authors:** Jane D. McLeod, Elizabeth M. Anderson

**Affiliations:** Department of Sociology, Indiana University, Bloomington, IN USA

**Keywords:** Autism spectrum disorder, Autistic traits, Postsecondary education, Social interactions, Academic outcomes, Surveys and questionnaire

## Abstract

This study evaluated the association of autistic traits (RAADS-14) with academic and social outcomes among college students using data from an online survey (N = 2,736). In the academic domain, the total trait score and all subscale scores (mentalizing deficits, social anxiety, sensory reactivity) were associated with course failure and academic difficulties independent of an autism diagnosis; the total score and mentalizing deficits also predicted lower grade point average (GPA). In the social domain, the total trait score and subscale scores were associated with lower odds of having a confidant, lower friendship quality, and higher odds of social exclusion. Subgroup analyses revealed that autistic traits had more consistently negative associations with social outcomes for students without an autism diagnosis than for students with a diagnosis. Associations were also more often significant for women than men. These results support the development of programs and services for students with autistic traits regardless of diagnostic status.

## Autistic Traits and College Adjustment

Research interest in the experiences of autistic college students has grown over the past ten years (e.g., Anderson & Butt 2017; Cox et al., 2017; Gelbar et al., 2015; Shattuck et al., 2012; Wei et al., 2014; White et al., 2011). This interest derives, in part, from evidence that autistic high school students increasingly aspire to a college education and enroll in college at higher rates than in the past (Anderson et al., 2016; Wagner et al., 2005; Wehman et al., 2014) yet do less well in college and graduate at much lower rates than neurotypical students (e.g., Anderson et al., 2016; Sanford et al., 2011; Wehman et al., 2014). Several explanations have been offered for these low graduation rates, including poor college preparation, impaired executive functioning, low reading proficiency, pragmatic language difficulties, and limited social engagement (e.g., Dijkhuis et al., 2020; Elias & White, 2018; Gelbar et al., 2015; Trevisan & Birmingham, 2015).

Studies that evaluate these explanations typically focus exclusively on autistic students or compare autistic students to neurotypical students based on a binary classification of autism (e.g., Casagrande et al., 2020; Elias & White, 2018; Gurbuz et al., 2019; Jackson et al., 2018; Lei et al., 2019). We take a different approach in this analysis by asking whether autistic traits predict outcomes independent of the diagnosis and whether the associations differ for students with and without an autism diagnosis and by gender.

Our interest in autistic traits derives from two related lines of research. The first provides evidence that there is significant variation in autistic traits in both autistic and neurotypical samples (e.g., De Groot & Van Strien 2017; Stevenson & Hart, 2017). By implication, binary autism diagnoses do not adequately represent the full range of autistic traits in the population. The second, and related, line of research concerns barriers to receiving an autism diagnosis. Delays in diagnosis remain common, especially for persons without cognitive impairments (Hosozawa et al., 2020; Lewis, 2017), racial and ethnic minority youth, and females (Daniels & Mandell, 2014; Jo et al., 2015; Rivet & Matson, 2011). The high rates of psychiatric comorbidity (Mannion & Leader, 2013) further complicate the diagnostic process (Mazzone et al., 2012). As a result of these diagnostic challenges, a sizable number of college students with high levels of autistic traits may have never received a diagnosis.

To provide a more holistic evaluation of the role of autism in college success, we evaluate the association of an autism diagnosis and dimensional measures of autistic traits with two sets of outcomes—academic success and social relationships—in a probability sample of college undergraduate students. Our focus on academic success aligns our analysis with growing interest in barriers to college graduation for autistic students (e.g., C. Anderson et al., 2016; Gelbar et al., 2015; Jackson et al., 2018; McLeod et al., 2019; Shattuck et al., 2012; White et al., 2011). Our interest in social relationships builds from evidence that autistic students face challenges establishing and maintaining meaningful interpersonal relationships in college settings (Casagrande et al., 2020; Gurbuz et al., 2019) and that social relationships and sense of belonging are important determinants of academic success for college students in general (Hurtado & Carter, 1997; Kuh, 2008; Museus et al., 2017; Tinto, 1998) as well as for autistic students specifically (Bailey et al., 2019; Freeth et al., 2012; VanBergeijk et al., 2008).

### The experiences of autistic college students

Evidence of the challenges autistic college students experience comes primarily from the National Longitudinal Transition Study – 2 (NLTS-2; Newman et al., 2011; Sanford et al., 2011) and from interview studies with small samples of autistic students (e.g., Anderson & Butt 2017; Cox et al., 2017). Studies based on the NLTS-2 show that autistic youth have lower rates of employment, vocational education, and college enrollment than youth with other disabilities (Shattuck et al., 2012). Moreover, autistic students who do enroll in college are disproportionately likely to attend 2-year rather than 4-year institutions (Wei et al., 2014).

Interview and small survey studies complement the NLTS-2 by adding direct reports from students themselves about the challenges they experience and the strategies they adopt to succeed. For example, autistic students report strategic disclosure of their diagnosis so that they receive academic support while avoiding stigma (disclosing to school staff when accommodations are needed but only disclosing to peers when circumstances require; Anderson & Butt 2017; Bolourian et al., 2018; Cox et al., 2017; Cullen, 2015; Harn et al., 2020; Lambe et al., 2019). Autistic students also identify gaps in support, particularly in relation to social relationships, emotional health, and career services, that reduce the quality of their educational experience (Gelbar et al., 2015). Although rich in detail, these studies are based on small samples of students, often from single institutions, which limits the generalizability of the results.

Recently, several larger surveys have been conducted that provide more representative data on the experiences of autistic students. White and colleagues (2011) conducted a survey of 667 college students, 13 of whom scored above the clinical threshold for autism on the Autism Spectrum Quotient (AQ; Baron-Cohen et al., 2001). Students with high AQ scores reported high levels of social anxiety, verbal aggression, and low levels of satisfaction with college, relative to students with low scores. Jackson and colleagues (2018) conducted a national online survey of college students with an autism diagnosis. Their sample of 56 respondents reported satisfaction with their academic and social lives but also high rates of depression, social anxiety, and loneliness. Because that study did not include a comparison group, it is not possible to determine how the experiences of these autistic students compared to neurotypical students at their institutions. Using a large national sample of college freshmen, Sturm & Kasari (2019) observed that autistic students felt less academically competent and reported lower levels of interpersonal competence than other students. Finally, McLeod and colleagues (2019) reported results from a large survey study of college students that included both students who had received autism diagnoses and a comparison group of students who had not. They found that students with an autism diagnosis reported poorer academic performance, social relationships, and physical and mental health compared to students without a diagnosis.

As these descriptions highlight, when comparisons between autistic students and neurotypical students are presented, they are typically based on binary indicators of autism: diagnosis v. not; above a clinical threshold v. not. Given barriers to accurate autism diagnosis (e.g., Daniels & Mandell 2014; Jo et al., 2015; Rivet & Matson, 2011), diagnostic indicators alone do not necessarily identify all students with autistic traits in college populations. Perhaps as important, relying on diagnostic indicators fails to acknowledge the challenges that autistic traits may present for college students whose traits do not reach a clinical threshold for diagnosis. Moreover, relying on diagnostic indicators is problematic from a methodological perspective inasmuch as the presence of students with autistic traits in comparison samples biases downward estimates of the relevance of autism for college experiences. This holds true even for non-diagnostic binary indicators to the extent that they miss the full range of autistic traits that may have implications for college success.

Drawing from these limitations, we use data from a probability sample of college students to answer three research questions:

- Do autistic traits predict academic and social outcomes independent of an autism diagnosis?
- Do autistic traits predict outcomes for students with and without a diagnosis of autism?
- Based on evidence that autistic traits and autism diagnoses vary by gender, do the associations of autistic traits with outcomes differ for male and female college students?

### The association of autistic traits with outcomes

The continuum of autistic traits has been conceptualized by some researchers as the broad autism phenotype, a reference to subclinical levels of autistic traits among persons who are not diagnosed with an autism spectrum disorder (Bolton et al., 1994; Piven & Palmer, 1999). Although some studies have challenged the reliability and validity of some measures of the broad autism phenotype (Stevenson & Hart, 2017; Agelink van Rentergem et al., 2019), evidence nonetheless supports the conclusion that there is substantial variation in autistic traits in the general population (Baron-Cohen et al., 2001; Constantino & Todd, 2003, 2005; De Groot & Van Strien, 2017; Wainer et al., 2011) and that this variation is associated with a range of social challenges (Sasson et al., 2012).

Much of the research on the association of autistic traits with social outcomes has been conducted with college student populations (e.g., Reed et al., 2016). For example, in a sample of college undergraduates, student scores on the Autism Spectrum Quotient were negatively associated with the number of reported friendships and length of best friendship (Jobe & White, 2007). In a study of undergraduate student friendship dyads, Wainer and colleagues (2013) found that higher scores on the Broad Autism Phenotype Questionnaire (BAPQ; Hurley et al., 2007) were associated with less desire for close relationships, greater loneliness, lower quality friendships, and more negative interactions. In complement, Stice & Lavner (2019) observed a significant association between scores on the BAPQ and internalizing symptoms that was attributable to variation in social connectedness and loneliness in a sample of undergraduate students. (See also Faso et al., 2016; and Sasson et al., 2013). While these studies confirm the relevance of autistic traits for social outcomes among college students, we are not aware of comparable studies that evaluate the association of autistic traits with *academic* outcomes in general student samples.

The extension to academic outcomes follows logically from evidence that social relationships importantly influence college student success. Education researchers have established that college students who feel connected to their institutions and who have positive and meaningful relationships with faculty, staff, and other students are more academically successful and more likely to persist in postsecondary education than students who lack such relationships (Astin, 1999; Tinto, 1998). Despite the logical connection between social and academic struggles, direct evidence for the association of autistic traits with academic outcomes is lacking. Inasmuch as academic support services are largely targeted to students who provide evidence of a medical diagnosis, knowing whether autistic traits are associated with academic outcomes independent of a diagnosis could inform how services are provided in future.

We hypothesize that the associations of autistic traits with outcomes are specific, i.e., that different traits matter for different outcomes. The measure we use, the RAADS-14 (Ritvo Autism and Asperger Diagnostic Scale-Revised-14; Eriksson et al., 2013), has three subscales: mentalizing deficits, social anxiety, sensory reactivity. Mentalizing deficits encompass challenges in understanding the intentions of others as well as rigidity. Social anxiety refers to challenges interacting with others in social or group situations. Sensory reactivity encompasses sensory sensitivities. Given the nature of these symptom subgroups, we expect mentalizing deficits to be more closely associated with academic outcomes than social anxiety and sensory reactivity, and mentalizing deficits and social anxiety to be more closely associated with social outcomes than sensory reactivity.

### Differences based on autism diagnosis

Although studies have considered the predictive power of autistic traits in samples of young adults both with and without an autism diagnosis, results are inconsistent and vary by specific outcome; few studies have directly compared associations in the two groups. Bailey and colleagues (2019) reported that autistic traits were not associated with life satisfaction in a sample of college students identified by disability services. In contrast, Dijkhuis and colleagues (2020) reported that a continuous measure of autism severity and measures of executive functioning were associated with academic progress among university students with diagnosed autism. Trevisan & Birmingham (2015) estimated the association of three dimensions of the BAPQ—aloof personality, rigid personality, and pragmatic language difficulties—with an omnibus measure of academic and social adjustment in a sample of 134 neurotypical college students. Of the dimensions, pragmatic language difficulties were more strongly associated with adjustment. Lei et al., (2020) found that autistic traits were associated with lower socialization adjustment and with lower levels of overall adjustment to college for typically developing students but not for autistic students. Taken as a whole, although the evidence is limited, these results suggest that autistic traits are more strongly associated with academic progress than with satisfaction and, perhaps, more strongly associated with outcomes for typically developing students than for autistic students.

### Gender differences

Our interest in gender derives from evidence that autism spectrum disorders are much more commonly diagnosed among boys than girls and are underdiagnosed in girls, with the gender difference being especially pronounced in populations without intellectual disability (Siklos & Kerns, 2007; see Rivets & Matson 2011 for a review). If autism is underdiagnosed in girls, we would expect to find more female, than male, college students who have autistic traits that have not been formally recognized. By implication, autistic traits should be more strongly associated with academic and social outcomes for young women than young men, because autistic traits are more independent of diagnostic status for young women, i.e., they have greater independent predictive power.

The social challenges associated with autism may also be less compatible with friendship expectations for women than for men. Women tend to value intimacy and reciprocity in friendships and to form friendships based on emotional sharing whereas men’s friendships are based more on sharing of activities and interests (Aukett et al., 1988; Felmlee et al., 2012; Hall, 2011; Koenig & Tsatsanis, 2005). As a result, autistic young women may experience more challenges making and maintaining friendships in college environments than autistic young men (Kreiser & White, 2014). In support of this expectation, prior research finds that autistic adolescent girls report more conflict in their friendships than autistic adolescent boys (Sedgewick et al., 2019). At the same time, some research suggests that adolescent girls may be better able than adolescent boys to mask their autistic behavior in interpersonal interactions and, as a result, may be able to avoid the social repercussions of their autism (Lai et al., 2015). This may explain why some studies find that observed gender differences in friendship conflict among autistic adolescents reflect more general gender differences in friendship (Sedgewick et al., 2019). We extend prior research by examining gender differences in the association of autistic traits with social outcomes in a college sample.

The current study is part of a larger project that draws from general research on the predictors of college success to analyze the specific experiences of autistic students. Our current focus on autistic traits extends prior research on college success and on autism by explicitly evaluating whether autistic traits affect college adjustment over and above an autism diagnosis. We consider variation across theoretically relevant subgroups of the student population, and account for demographic and student characteristics that may differ by autism diagnosis and/or autistic traits to bolster confidence that any observed differences in outcomes are not due to confounding.

## Methods

The data for the analysis come from an online survey of college students that was fielded at 14 2-year and 4-year postsecondary institutions in Indiana in spring 2017. The survey was programmed in Qualtrics and implemented by the Center for Survey Research at Indiana University. The Indiana University Institutional Review Board reviewed and approved all study procedures (protocol 1606175874).

### Sample recruitment

All public 2-year and 4-year post-secondary institutions in the state were invited to participate in the study (N = 26). In all, 14 institutions participated including six of eleven regions of the statewide community college system (we count regional campuses as separate institutions). Participating institutions varied in their selectivity and size, with total undergraduate student populations ranging from approximately 3,000 to 30,000. Each participating institution was asked to contribute two samples of students: (1) all students who were currently registered for disability accommodations based on autism (the “registered” sample) and (2) a probability sample of currently enrolled, degree-seeking undergraduate students (the “general sample”). All participating institutions agreed to provide a registered sample. Ten institutions also provided a general sample. The general samples allowed us to capture students who had been diagnosed on the autism spectrum but who were not registered for accommodations and to compare students on the spectrum to their neurotypical peers.

The Indiana University Center for Survey Research recruited students in the general sample to the study via emails that introduced the study and provided links to the survey. The emails described the study as concerned with “student experiences in coursework, social life, and beyond to learn more about what makes college special for some students and not so special for others.” Recruitment emails to the general sample were personalized and included a personalized link to the survey. With the exception of one institution which asked us to recruit students in the registered sample directly, to protect the privacy of those students, institutions sent recruitment emails directly to registered students. The recruitment email for the registered sample was the same as that for the general sample except that we identified a special interest in “the experiences of students on the autism spectrum.”

For both samples, the initial invitation was followed by two reminder messages, sent roughly 7–10 days later. In the early stages of the survey, participants in the general sample were offered a $5 gift card and entry into a drawing for $500 to complete the survey; participants in the registered sample were offered a $10 gift card and the same drawing entry. In order to increase response rates, the respondent incentives were increased to $10 and $20, respectively, at the second and third invitations.

Students who chose to participate in the survey clicked on the survey link in their email which directed them to the informed consent statement. After reading the statement, they had the option of consenting to participate or declining participation. Those who chose to participate typically completed the online survey at that time.

Institutions provided basic demographic information (age, race/ethnicity, gender, domestic v. international status) for their general samples; institutions did not provide the same information for the registered samples. By comparing the demographic characteristics of participants to those of their institutions, we were able to evaluate bias in the sample. Although there is some variation across institutions, consistent with prior research on online surveys (Sax et al., 2008), women and white respondents tend to be slightly overrepresented among participants in the general sample.

### Survey Questionnaire

The investigators developed the questionnaire for this study based on existing national surveys of young adults and college students, including the National Survey of Student Engagement (an annual national survey of the quality of undergraduate education; Kuh 2009), the Wabash National Study of Liberal Arts Education (a longitudinal, national survey of undergraduates at liberal arts institutions; Blaich & Wise 2011), and the National Longitudinal Study of Adolescent to Adult Health (Harris & Udry, 2018). We chose items from national surveys because they have been extensively validated and so that we could compare our results to national norms for undergraduate college students. The draft questionnaire was shared with members of a campus autism support group for their feedback before being pretested. Based on this feedback, the informed consent statement was reorganized to present less information per computer screen, and response options for some questions were simplified. The questionnaire was then pretested with a sample of college students from a non-participating institution.

Survey questions covered a range of topics related to college experiences, including prior educational background, motivations for attending college, academic challenges, course-taking and classroom behaviors, feelings of belonging, social relationships, and health. Participants who reported a diagnosis of autism also answered questions on whether they disclosed their diagnosis to their college and on autism identity. The sources of questions in specific topic areas are described in greater detail below.

### Response rate and sample size

In total, 3,216 students completed the survey, of whom 78 were in the registered sample. Using AAPOR’s Response Rate 1 (the “minimum response rate,” American Association of Public Opinion Research, 2016), the response rate for the general sample was about 14%, with response rates for individual institutions ranging from 6.32 to 25.45%. The response rate for the registered sample was about 15%, with response rates for individual institutions ranging from 1.10 to 31.71%.

We restrict our analysis to students ages 34 and younger, 88% of our sample, based on evidence that over 90% of full-time or part-time undergraduate students at public institutions fall into that age range (McFarland et al., 2019).^1^ This leaves us with a sample of 2,820 students before accounting for missing data. Although we chose the age restriction to capture the typical age range for college students at public institutions, the choice was inconsequential: the pattern of results was the same for youth ages 24 and younger (a range that captures about 60% of postsecondary undergraduate students at public institutions) and for the full sample (ages 18–76).

### Measures

#### Autism diagnosis

Participants responded to a series of questions about diagnoses they had received, including “an autism spectrum disorder.” We assume that students who have been offered academic accommodations for autism by their schools (the registered group) had to provide medical documentation of the need. Therefore, for purposes of this analysis, participants were defined as having an autism diagnosis if they reported having ever received a diagnosis of an autism spectrum disorder or if they were in the registered sample.

#### Autistic traits

We measured autistic traits with the RAADS-14, a short form of the Ritvo Autism and Asperger Diagnostic Scale – Revised, a screening instrument designed to identify undiagnosed cases of ASD (Eriksson et al., 2013). Recent research suggests that the RAADS-14 and AQ-10 (short form of the Autism Spectrum Quotient; Baron-Cohen et al., 2001) perform equally well in identifying cases of autism, with the AQ-10 having somewhat higher specificity and the RAADS-14 having somewhat higher sensitivity (Sizoo et al., 2015) The RAADS-14 is highly correlated with the Adult Autism Subthreshold Spectrum (Dell’Osso et al., 2017) and has been found to have reasonable psychometric properties (Baghdadli et al., 2017).

The RAADS-14 includes 14 items for each of which respondents report whether it was “true now and when I was young” (= 3), “true only now” (= 2), “true only when I was younger than 16” (= 1) and “never true” (= 0). Item values are summed to arrive at a total score and subscale scores for mentalizing deficits, social anxiety, and sensory reactivity. Correlations among the subscale scores ranged from 0.45 to 0.59. Table 1 presents the items in each subscale. As noted, mentalizing deficits encompass challenges in understanding the intentions of others and rigidity, social anxiety refers to challenges interacting with others in social or group situations, and sensory reactivity encompasses sensory sensitivities.

**Table 1:** RAADS-14 Items by Subscale (Eriksson et al., 2013)

Subscale	RAADS-14 Item
Mentalizing deficits	I take things too literally, so I often miss what people are trying to say
	It is difficult for me to understand how other people are feeling when we are talking
	When talking to someone, I have a hard time telling when it is my turn to talk or to listen
	It is difficult to figure out what other people expect of me
	It can be very hard to read someone’s face, hand, and body movements when we are talking
	I focus on details rather than the overall idea
	I get extremely upset when the way I like to do things is suddenly changed
Social Anxiety	It is very difficult for me to work and function in groups
	I often don’t know how to act in social situations
	I can chat and make small talk with people *reversed statement
	How to make friends and socialize is a mystery to me
Sensory Reactivity	Some ordinary textures that do not bother others feel very offensive when they touch my skin
	When I feel overwhelmed by my senses, I have to isolate myself to shut them down
	Sometimes I have to cover my ears to block out painful noises (like vacuum cleaners or people talking too much or too loudly)

The RAADS-14 has strong internal reliability in this sample (α = 0.85). It also discriminates well between students with and without ASD diagnoses. Students with ASD diagnoses scored significantly higher (mean = 25.63) on the RAADS-14 than students without ASD diagnoses (mean = 10.23; t= -16.44, p < .001). Using the recommended cut-point of 14 for identifying ASD, students who scored above the cut-point were significantly more likely than those who scored at or below the cut-point to report an ASD diagnosis (9.47% vs. 0.38%; χ^2^ = 154.79, p < .001).

#### Outcomes

We considered three self-reported academic outcomes: grade point average (GPA); whether the student had ever failed a course at their current institution; and academic difficulties. Students reported their cumulative GPA from their most recent semester on a scale from 0.0 to 4.0. Course failure was based on the following question: “Since entering this institution, which of the following have you done?” for which one follow-up was “failed one or more courses.” Academic difficulties was a summated scale based on a question series from the National Survey of Student Engagement that began “(d)uring the current school year, how difficult (if at all) have the following been for you?” Items included “learning course material,” “understanding what your professors expect of you academically,” “managing your time effectively,” “maintaining a routine,” and “staying organized.” For each item, respondents gave answers from 1=“not at all” to 4=“very.” We averaged responses across the items to yield a final score ranging from 1 to 4.

We also considered three social outcomes: whether the respondent has a confidant; their assessment of their friendship experiences on campus; and social exclusion. Our measure of access to a confidant was based on a single item: “Is there anyone you can really open up to about your most private feelings without having to hold them back?” This item is a common indicator of social support, derived from the National Comorbidity Survey (Kessler, 1994).

Our measure of friendship experiences was a summated scale based on four statements, for which respondents were asked how strongly they agreed or disagreed on a 4-point scale: “Since coming to this institution, I have developed close personal relationships with other students.” “The student friendships I have developed at this institution have been personally satisfying.” “I wish I had more close personal relationships with other students.” “It has been difficult for me to meet and make friends with other students.” The items derive from the Wabash National Study of Liberal Arts Education (Blaich & Wise, 2011). Individual items were coded so that a high value (4) represents greater satisfaction with friendships and were then averaged, yielding a value from 1 to 4.

To measure social exclusion, we used an item from Schäfer et al.’s (2004) *Retrospective Bullying Questionnaire*: “Have other students at this institution ever told lies or nasty rumors about you, or deliberately excluded you from social groups or activities?”

### Demographic and enrollment characteristics

Participants responded to questions about several demographic and enrollment characteristics, including their current age, race/ethnicity, gender identity (man, woman, other [intersex, transgender, and other volunteered responses]), sexual identity (heterosexual/straight, gay, lesbian, bisexual, queer, asexual, questioning or unsure, other), marital status, and parents’ highest level of education (coded here as a dichotomy: less than a bachelor’s degree v. bachelor’s degree or higher). Students also reported their year in school (1st, 2nd, 3rd, 4th or higher), and full-time/part-time student status. Because of small sample sizes in the autism subsample, we combined all non-white racial/ethnic groups into a single “non-white” category for race and combined all non-heterosexual groups into a single comparison category for sexual identity. Many of these characteristics are associated both with autism and with academic or social outcomes, recommending their use as controls in our models.

Several variables had missing values, with the largest number of missing values being for the items for demographics: these questions appeared relatively late in the survey. We omitted respondents who had missing values on any of the analysis variables. Of the 2,820 students who were eligible for this analysis based on age, N = 2,736 had valid values on all analysis variables. Sample sizes vary slightly across dependent variables to provide the maximum possible sample size for each model.

### Statistical analysis

We conducted our analysis in three stages corresponding to our three research questions. First, to answer the question of whether autistic traits predict outcomes independent of an autism diagnosis, we estimated a series of regression models with the first including only the indicator for autism diagnosis and the control variables and subsequent models adding the autistic traits scale and subscales. Second, to determine whether autistic traits predict outcomes for students with and without an autism diagnosis, we estimated the association of autistic traits with outcomes separately in those two subgroups. Finally, we re-estimated both sets of models separately for male and female students to determine whether there are gender differences in the association of autistic traits with academic and social outcomes.

The autistic trait scale and subscales were standardized before inclusion in all models. Logistic regression models were used for binary outcomes (failed a course, confidant, social exclusion). Ordinary least squares regression models were used for the other outcomes (GPA, academic difficulties, friendship quality). We used sample weights to account for differential probability of selection across institutions, and robust standard errors were estimated to account for clustering of students within institutions. All analyses were conducted in Stata 16.1.

## Results

Table 2 presents descriptive statistics for the main analysis variables including the controls. In the sample as a whole, the average GPA was fairly high (3.26). At the same time, almost one-quarter of the students had failed a class at their current institution. The average level of reported academic difficulties was low at 2.16, just above “a little bit difficult.” The sample presented favorable social outcomes overall, with a high percentage (80.40%) reporting a confidant, an average friendship relationship score of 2.74, and a low rate of social exclusion (9.89%). Of the students in the analysis sample, 90 were coded as having an autism diagnosis. The average score on the RAADS-14 was 10.74. The median is 9, indicating that most students scored far below the recommended clinical cutoff of 14.

**Table 2:** Descriptive Statistics for Analysis Variables

	N/Mean	Proportion/ Standard Deviation
*Respondent Characteristics*		
Age	21.33	3.37
Race		
White	2,070	75.66%
Black	164	5.99%
Hispanic	93	3.40%
Asian	248	9.06%
Other race	161	5.88%
Gender		
Male	994	36.42%
Female	1,703	62.40%
Other gender	32	1.17%
Marital Status		
Never married	2,485	90.83%
Currently married	199	7.27%
Previously married	52	1.90%
Sexual Identity		
Straight	2,398	87.65%
Gay	67	2.45%
Bisexual	151	5.52%
Other sexual identity	120	4.39%
Parent’s Education		
BA or higher	1,573	57.49%
Less than BA	1,163	42.51%
Autism Diagnosis		
Yes	90	3.30%
No	2,644	96.71%
Autism Scales		
Autistic traits scale	10.74	9.16
Mentalizing deficits scale	5.53	5.26
Social anxiety scale	3.00	3.06
Sensory reactivity scale	2.20	2.46
*Academic Characteristics*		
International Student		
Yes	177	6.47%
No	2,559	93.53%
Full-time Student		
Yes	2,368	86.55%
No	368	13.45%
Academic Year		
1st year	917	33.52%
2nd year	795	29.06%
3rd year	524	19.15%
4th year or higher	500	18.27%
Institution Type		
2-year	749	27.38%
4-year	1,987	72.62%
*Dependent Variables*		
GPA	3.26	0.57
Previously Failed Course		
Yes	630	23.16%
No	2,090	76.84%
Academic Difficulties	2.16	0.65
Confidant		
Yes	2,194	80.40%
No	535	19.60%
Friendship Quality	2.74	0.64
Social Exclusion		
Yes	270	9.89%
No	2,460	90.11%

Demographically, the sample was majority white and female, with the total percent of non-white students at about 25%. Most of the students in the sample were enrolled full-time and most attended 4-year institutions. The average respondent was in the second year at their institution.

### Do autistic traits predict outcomes independent of a diagnosis?

Our first research question asks whether autistic traits are associated with academic and social outcomes independent of an autism diagnosis. Table 3 presents the coefficients and odds-ratios (OR) from the relevant regression models for academic outcomes. The tables do not include the coefficients for the control variables for parsimony of presentation, although those variables were included in the models. For each outcome, Model 1 (M1) evaluates the association of the autism diagnosis with academic outcomes. Model 2 (M2) adds the total autistic trait score as a predictor. Models 3–5 replace the total autistic trait score with its components: mentalizing deficits; social anxiety; and sensory reactivity.

**Table 3:** Coefficients from Regressions of Academic Outcomes on Autism Indicators

GPA	M1	M2	M3	M4	M5
Autism diagnosis	-0.19^*^ (0.07)	-0.13 (0.07)	-0.12 (0.07)	-0.18^*^ (0.07)	-0.16^*^ (0.07)
Autistic traits	−	-0.04^**^ (0.01)	−	−	−
Mentalizing deficits	−	−	-0.04^**^ (0.01)	−	−
Social anxiety	−	−	−	-0.01 (0.01)	−
Sensory reactivity	−	−	−	−	-0.02 (0.01)
N=	2,579	2,576	2,570	2,575	2,574
**Failed a course**	**M1**	**M2**	**M3**	**M4**	**M5**
Autism diagnosis	1.37 (0.33)	1.07 (0.25)	1.06 (0.24)	1.23 (0.28)	1.26 (0.31)
Autistic traits	−	1.16^***^ (0.03)	−	−	−
Mentalizing deficits	−	−	1.18^***^ (0.04)	−	−
Social anxiety	−	−	−	1.11^***^ (0.03)	−
Sensory reactivity	−	−	−	−	1.07^*^ (0.03)
N=	2,719	2,715	2,707	2,713	2,712
**Academic difficulties**	**M1**	**M2**	**M3**	**M4**	**M5**
Autism diagnosis	0.18^**^ (0.06)	-0.11 (0.06)	-0.08 (0.07)	0.03 (0.06)	0.05 (0.06)
Autistic traits	−	0.18^***^ (0.01)	−	−	−
Mentalizing deficits	−	−	0.16^***^ (0.01)	−	−
Social anxiety	−	−	−	0.14^***^ (0.01)	−
Sensory reactivity	−	−	−	−	0.11^***^ (0.01)
N=	2,731	2,729	2,721	2,727	2,726

Notes: Coefficients and standard errors (SE) for GPA and academic difficulties are from OLS regressions. Coefficients and standard errors for course failure are exponentiated, from logistic regressions. *p < .05; **p < .01; ***p < .001

Before accounting for autistic traits, an autism diagnosis was significantly associated with GPA and with academic difficulties but not with course failure: students with an autism diagnosis reported lower GPAs (β = - 0.19, p < .05) and more academic difficulties (β = 0.18, p < .01) than students without an autism diagnosis. The total autistic trait score and several subscale scores were also significantly associated with academic outcomes independent of an autism diagnosis. The total score and mentalizing deficits predicted lower GPA (β=-0.04, p < .01 for both), and the total score and all subscale scores were associated with a higher probability of failing a course (OR = 1.07 to OR = 1.18) and higher levels of academic difficulties (β = 0.11 to β = 0.18). With the addition of the autistic trait scores to the model, an autism diagnosis was no longer associated with academic difficulties. In sum, for academic outcomes, autistic trait scores were associated with poorer performance independent of the autism diagnosis itself.

Table 4 presents the coefficients from comparable regression models predicting social outcomes in the total sample. For social outcomes as well, the associations with trait scores were significant independent of the autism diagnosis. Students with an autism diagnosis had significantly lower odds of reporting a confidant and higher odds of social exclusion than students without a diagnosis (OR = 0.45, p < .01 and OR = 2.54, p < .01, respectively), but there was no difference in friendship quality. In contrast, the total trait score and all subscale scores were associated with a lower likelihood of having a confidant (OR = 0.67 to 0.90), lower friendship quality (β=-0.08 to -0.22), and a higher likelihood of being socially excluded (OR = 1.18 to OR = 1.45). These results provide support for our general hypothesis that autistic traits predict academic and social outcomes independent of an autism diagnosis, if not always for our hypotheses about the associations of specific sets of traits with specific outcomes.

**Table 4:** Coefficients from Regressions of Social Outcomes on Autism Indicators

Confidant	M1	M2	M3	M4	M5
Autism diagnosis	0.45^**^ (0.11)	0.76 (0.25)	0.69 (0.24)	0.67 (0.19)	0.51^*^ (0.14)
Autistic traits	−	0.71^***^ (0.05)	−	−	−
Mentalizing deficits	−	−	0.75^***^ (0.06)	−	−
Social anxiety	−	−	−	0.67^***^ (0.04)	−
Sensory reactivity	−	−	−	−	0.90^*^ (0.04)
N=	2,728	2,724	2,716	2,722	2,721
**Friendship Quality**	**M1**	**M2**	**M3**	**M4**	**M5**
Autism diagnosis	-0.14 (0.11)	0.13 (0.08)	0.04 (0.09)	0.10 (0.07)	-0.04 (0.10)
Autistic traits	−	-0.17^***^ (0.01)	−	−	−
Mentalizing deficits	−	−	-0.11^***^ (0.01)	−	−
Social anxiety	−	−	−	-0.22^***^ (0.02)	−
Sensory reactivity	−	−	−	−	-0.08^***^ (0.01)
N=	2,723	2,721	2,714	2,719	2,718
**Social Exclusion**	**M1**	**M2**	**M3**	**M4**	**M5**
Autism diagnosis	2.54^**^ (0.87)	1.44 (0.57)	1.44 (0.56)	2.14 (0.83)	1.76 (0.60)
Autistic traits	−	1.43^***^ (0.10)	−	−	−
Mentalizing deficits	−	−	1.45^***^ (0.10)	−	−
Social anxiety	−	−	−	1.18^*^ (0.08)	−
Sensory reactivity	−	−	−	−	1.33^***^ (0.08)
N=	2,728	2,725	2,717	2,723	2,722

Notes: Coefficients and standard errors (SE) for friendship quality are from OLS regressions. Coefficients and standard errors for confidant and social exclusion are exponentiated, from logistic regressions. *p < .05; **p < .01; ***p < .001

### Do the associations differ by autism diagnosis?

With these basic results in hand, we considered the extent to which the associations of autistic traits with academic and social outcomes differed based on autism diagnostic status. Tables 5 and 6 present the coefficients from regression models for academic and social outcomes, respectively, for the samples with and without an autism diagnosis. Here, Models 1–4 (M1-M4) represent models that included the total autistic trait score, mentalizing deficits, social anxiety, and sensory reactivity, respectively. In each table, Panel A presents the results for the students with an autism diagnosis, Panel B presents the results for the students without an autism diagnosis.

**Table 5:** Coefficients from Regressions of Academic Outcomes on Autism Indicators for Students With and Without an Autism Diagnosis

	Panel A: Sample with Autism Diagnosis (N = 90)				Panel B: Sample without Autism Diagnosis (N = 2,644)			
**GPA**	**M1**	**M2**	**M3**	**M4**	**M1**	**M2**	**M3**	**M4**
Autistic traits	-0.06 (0.09)	−	−	−	-0.03^*^ (0.01)	−	−	−
Mentalizing deficits	−	0.02 (0.07)	−	−	−	-0.04^**^ (0.01)	−	−
Social anxiety	−	−	-0.11 (0.08)	−	−	−	-0.01 (0.01)	−
Sensory reactivity	−	−	−	-0.07 (0.06)	−	−	−	-0.02 (0.01)
**Failed a Course**	**M1**	**M2**	**M3**	**M4**	**M1**	**M2**	**M3**	**M4**
Autistic traits	1.71^*^ (0.42)	−	−	−	1.13^***^ (0.03)	−	−	−
Mentalizing deficits	−	1.41 (0.41)	−	−	−	1.15^***^ (0.03)	−	−
Social anxiety	−	−	1.69^*^ (0.41)	−	−	−	1.08^**^ (0.03)	−
Sensory reactivity	−	−	−	1.45 (0.37)	−	−	−	1.06 (0.03)
**Academic difficulties**	**M1**	**M2**	**M3**	**M4**	**M1**	**M2**	**M3**	**M4**
Autistic traits	0.37^***^ (0.09)	−	−	−	0.16^***^ (0.01)	−	−	−
Mentalizing deficits	−	0.30^**^ (0.09)	−	−	−	0.15^***^ (0.01)	−	−
Social anxiety	−	−	0.26^**^ (0.07)	−	−	−	0.13^***^ (0.01)	−
Sensory reactivity	−	−	−	0.28^*^ (0.10)	−	−	−	0.10^***^ (0.01)

Notes: Coefficients and standard errors (SE) for GPA and academic difficulties are from OLS regressions. Coefficients and standard errors for course failure are exponentiated, from logistic regressions. Sample sizes vary slightly from missingness in the dependent variables and autism scales. *p < .05; **p < .01; ***p < .001

**Table 6:** Coefficients from Regressions of Social Outcomes on Autism Indicators for Students Sith and Without an Autism Diagnosis

	Panel A: Sample with Autism Diagnosis (N = 90)				Panel B: Sample without Autism Diagnosis (N = 2,644)			
**Confidant**	**M1**	**M2**	**M3**	**M4**	**M1**	**M2**	**M3**	**M4**
Autistic traits	1.65^**^ (0.31)	−	−	−	0.68^***^ (0.05)	−	−	−
Mentalizing deficits	−	1.46 (0.30)	−	−	−	0.73^***^ (0.06)	−	−
Social anxiety	−	−	1.21 (0.27)	−	−	−	0.65^***^ (0.03)	−
Sensory reactivity	−	−	−	1.76^*^ (0.41)	−	−	−	0.85^***^ (0.04)
**Friendship Quality**	**M1**	**M2**	**M3**	**M4**	**M1**	**M2**	**M3**	**M4**
Autistic traits	-0.14 (0.10)	−	−	−	-0.16^***^ (0.01)	−	−	−
Mentalizing deficits	−	-0.06 (0.08)	−	−	−	-0.10^***^ (0.01)	−	−
Social anxiety	−	−	-0.26^*^ (0.09)	−	−	−	-0.21^***^ (0.02)	−
Sensory reactivity	−	−	−	0.00 (0.11)	−	−	−	-0.08^***^ (0.01)
**Social Exclusion**	**M1**	**M2**	**M3**	**M4**	**M1**	**M2**	**M3**	**M4**
Autistic traits	0.85 (0.28)	−	−	−	1.41^***^ (0.10)	−	−	−
Mentalizing deficits	−	1.29 (0.47)	−	−	−	1.43^***^ (0.10)	−	−
Social anxiety	−	−	0.37^*^ (0.18)	−	−	−	1.20^***^ (0.08)	−
Sensory reactivity	−	−	−	1.06 (0.35)	−	−	−	1.29^***^ (0.08)

Notes: Coefficients and standard errors (SE) for friendship quality are from OLS regressions. Coefficients and standard errors for confidant and social exclusion are exponentiated, from logistic regressions. Sample sizes vary slightly from missingness in the dependent variables and autism scales. *p < .05; **p < .01; ***p < .001

According to the results in Table 5, among students with an autism diagnosis, the total autistic trait score and several subscales were associated with some of the academic outcomes but not consistently. Specifically, students who scored higher on the total trait score were more likely to fail a course (OR = 1.71, p < .05) and reported more academic difficulties (β = 0.37, p < .001), but autistic traits were not associated with GPA. Students with higher scores on social anxiety were more likely to report having failed a course (OR = 1.69, p < .05) and students with high scores on mentalizing deficits, social anxiety, and sensory reactivity reported more academic difficulties (β = 0.30, p < .01, β = 0.26, p < .01, and β = 0.28, p < .05, respectively). None of the trait scores was associated with GPA.

Trait scores were more consistently associated with academic outcomes in the sample of students without an autism diagnosis. Higher total autistic trait scores were associated with a lower GPA (β=-0.03, p < .05), a higher probability of failing a course (OR = 1.13, p < .001), and higher academic difficulties (β = 0.16, p < .001). In addition, mentalizing deficits were associated with poor performance on all three academic outcomes, social anxiety was associated with a higher likelihood of course failure and higher levels of academic difficulties, and sensory reactivity was associated with higher levels of academic difficulties.

A brief review of the coefficients suggests that most of the differences in the associations based on autism diagnosis result from the relatively small size of the diagnosed sample and the relatively large standard errors for the coefficients. We tested the significance of the group differences in the coefficients using the suest (seemingly unrelated estimation) function in Stata. The positive associations of the trait scores with academic difficulties were all significantly stronger for students *with* an autism diagnosis than for students without a diagnosis. However, the other coefficient differences were not significant.

Turning to Table 6, results were somewhat more differentiated for social outcomes, with some unexpected results in the subsample of students with an autism diagnosis. Contrary to expectation, among students with an autism diagnosis, the total trait score and sensory reactivity were associated with a *higher* probability of having a confidant (OR = 1.65, p < .01, and OR = 1.76, p < .05, respectively), and social anxiety was associated with a lower probability of social exclusion (OR = 0.37, p < .05). More consistent with expectation, social anxiety was associated with lower friendship quality (β=-0.26, p < .05). Among students without an autism diagnosis, the total trait score and all subscale scores were significantly associated with social outcomes in the expected direction: a lower probability of having a confidant, lower friendship quality, and a higher probability of being socially excluded. The differences in coefficients for students with and without a diagnosis were significant for having a confidant but not for friendship quality or social exclusion.

### Do the associations differ by gender?

Our final research question considered gender differences in the association of an autism diagnosis and autistic traits with academic and social outcomes. Tables 7 and 8 present the relevant coefficients. Similar to the earlier discussion about autism diagnoses and autistic traits, Model 1 (M1) represents models that included only the autism diagnosis while Models 2–5 (M2-M5) represent models that included both the autism diagnosis along with the total trait score, mentalizing deficits, social anxiety, or sensory reactivity, respectively. In each table, Panel A presents the results for women and Panel B presents the results for men.

**Table 7:** Coefficients from Regressions of Academic Outcomes on Autism Indicators for Women and Men

	Panel C: Women (N = 1,703)					Panel D: Men (N = 1,026)				
**GPA**	**M1**	**M2**	**M3**	**M4**	**M5**	**M1**	**M2**	**M3**	**M4**	**M5**
Autism diagnosis	-0.23 (0.16)	-0.14 (0.14)	-0.15 (0.15)	-0.22 (0.15)	-0.17 (0.14)	-0.09 (0.07)	-0.07 (0.08)	-0.06 (0.08)	-0.08 (0.07)	-0.09 (0.08)
Autistic traits	−	-0.04^*^ (0.02)	−	−	−	−	-0.02 (0.01)	−	−	−
Mentalizing deficits	−	−	-0.05^**^ (0.02)	−	−	−	−	-0.02 (0.01)	−	−
Social anxiety	−	−	−	-0.01 (0.02)	−	−	−	−	-0.01 (0.02)	−
Sensory reactivity	−	−	−	−	-0.04^*^ (0.02)	−	−	−	−	0.00 (0.02)
**Failed a course**	**M1**	**M2**	**M3**	**M4**	**M5**	**M1**	**M2**	**M3**	**M4**	**M5**
Autism diagnosis	2.62^***^ (0.67)	1.84^*^ (0.51)	1.96^**^ (0.51)	2.35^**^ (0.62)	2.18^**^ (0.62)	0.83 (0.24)	0.71 (0.19)	0.69 (0.18)	0.75 (0.21)	0.83 (0.23)
Autistic traits	−	1.19^***^ (0.05)	−	−	−	−	1.12 (0.08)	−	−	−
Mentalizing deficits	−	−	1.20^***^ (0.05)	−	−	−	−	1.14 (0.09)	−	−
Social anxiety	−	−	−	1.08 (0.05)	−	−	−	−	1.12 (0.08)	−
Sensory reactivity	−	−	−	−	1.13^**^ (0.05)	−	−	−	−	1.00 (0.04)
**Academic difficulties**	**M1**	**M2**	**M3**	**M4**	**M5**	**M1**	**M2**	**M3**	**M4**	**M5**
Autism diagnosis	0.22^*^ (0.07)	-0.16 (0.07)	-0.12 (0.09)	0.03 (0.08)	0.05 (0.08)	0.13 (0.08)	-0.11 (0.08)	-0.08 (0.08)	0.00 (0.08)	-0.01 (0.09)
Autistic traits	−	0.18^***^ (0.01)	−	−	−	−	0.19^***^ (0.03)	−	−	−
Mentalizing deficits	−	−	0.17^***^ (0.02)	−	−	−	−	0.16^***^ (0.02)	−	−
Social anxiety	−	−	−	0.14^***^ (0.01)	−	−	−	−	0.16^***^ (0.02)	−
Sensory reactivity	−	−	−	−	0.11^***^ (0.01)	−	−	−	−	0.13^**^ (0.03)

Notes: Coefficients for GPA and academic difficulties are from OLS regressions. Coefficients for course failure are exponentiated, from logistic regressions. Sample sizes vary slightly from missingness in the dependent variables and autism scales

**Table 8:** Coefficients from Regressions of Social Outcomes on Autism Indicators for Women and Men

	Panel C: Women (N = 1,703)					Panel D: Men (N = 1,026)				
**Confidant**	**M1**	**M2**	**M3**	**M4**	**M5**	**M1**	**M2**	**M3**	**M4**	**M5**
Autism diagnosis	0.56 (0.29)	1.25 (0.61)	1.04 (0.56)	0.96 (0.43)	0.78 (0.42)	0.55 (0.23)	0.83 (0.42)	0.77 (0.39)	0.78 (0.38)	0.57 (0.26)
Autistic traits	−	0.67^***^ (0.06)	−	−	−	−	0.73^***^ (0.07)	−	−	−
Mentalizing deficits	−	−	0.72^***^ (0.07)	−	−	−	−	0.78^**^ (0.06)	−	−
Social anxiety	−	−	−	0.66^***^ (0.03)	−	−	−	−	0.63^***^ (0.08)	−
Sensory reactivity	−	−	−	−	0.81^*^ (0.07)	−	−	−	−	0.98 (0.07)
**Friendship Quality**	**M1**	**M2**	**M3**	**M4**	**M5**	**M1**	**M2**	**M3**	**M4**	**M5**
Autism diagnosis	-0.18 (0.13)	0.18 (0.11)	0.09 (0.10)	0.12 (0.11)	-0.04 (0.14)	-0.11 (0.09)	0.12 (0.07)	0.03 (0.09)	0.09 (0.06)	-0.02 (0.08)
Autistic traits	−	-0.17^***^ (0.01)	−	−	−	−	-0.17^***^ (0.02)	−	−	−
Mentalizing deficits	−	−	-0.11^***^ (0.01)	−	−	−	−	0.10^***^ (0.01)	−	−
Social anxiety	−	−	−	-0.21^***^ (0.02)	−	−	−	−	-0.24^***^ (0.03)	−
Sensory reactivity	−	−	−	−	-0.09^***^ (0.01)	−	−	−	−	-0.08^**^ (0.02)
**Social Exclusion**	**M1**	**M2**	**M3**	**M4**	**M5**	**M1**	**M2**	**M3**	**M4**	**M5**
Autism diagnosis	4.53^*^ (3.20)	2.53 (1.93)	2.67 (2.04)	3.97 (3.03)	3.01 (2.18)	2.38^**^ (0.79)	1.24 (0.54)	1.25 (0.55)	1.85 (0.70)	1.54 (0.59)
Autistic traits	−	1.32^***^ (0.12)	−	−	−	−	1.70^***^ (0.19)	−	−	−
Mentalizing deficits	−	−	1.34^***^ (0.11)	−	−	−	−	1.70^***^ (0.16)	−	−
Social anxiety	−	−	−	1.10 (0.09)	−	−	−	−	1.41^**^ (0.17)	−
Sensory reactivity	−	−	−	−	1.27^**^ (0.11)	−	−	−	−	1.44^***^ (0.14)

Notes: Coefficients for friendship quality are from OLS regressions. Coefficients for confidant and social exclusion are exponentiated, from logistic regressions. Sample sizes vary slightly from missingness in the dependent variables and autism scales

In general, both the autism diagnosis and the autistic traits scores were more strongly associated with outcomes for women than for men. For women, an autism diagnosis was associated with a higher likelihood of course failure (OR = 2.62, p < .001) and higher levels of academic difficulties (β = 0.22, p < .05) whereas for men the autism diagnosis was not associated with any academic outcomes. Turning to autistic traits, for women, the total trait score, mentalizing deficits, and sensory reactivity were all associated with lower GPA, a higher likelihood of course failure, and higher levels of academic difficulties, independent of the autism diagnosis; social anxiety was also associated with academic difficulties for women. In contrast, for men, the total score and all subscale scores were only associated with higher levels of academic difficulties. As for the comparison of students with and without an autism diagnosis, we tested the significance of gender differences in the coefficients. With the exception of course failure, most of the gender differences were not significant. For course failure, the associations of the autism diagnosis and sensory reactivity were significantly stronger for women than for men.

The pattern of associations for social outcomes was more similar for women and men. The autism diagnosis was significantly associated with social exclusion for both women and men. Although the odds-ratio was larger for women than for men, the difference was not significant, probably because of the relatively small number of young women with an autism diagnosis. In addition, the total trait score and most subscale scores were associated with a lower likelihood of having a confidant, lower friendship quality, and a higher likelihood of being socially excluded for both women and men. Despite these differences in the within-group results, none of the coefficients was significantly different by gender.

In sum, we observed that autistic traits consistently predict social and academic outcomes independent of an autism diagnosis. For academic outcomes, although the associations of autistic traits were more often significant among students without a diagnosis, the associations were not significantly different based on autism diagnostic status. For social outcomes, although most of the group differences were not statistically significant, the pattern of results suggests that autistic traits have more consistently negative implications for students without an autism diagnosis than for students with a diagnosis. The few significant gender differences we observed, for the associations of an autism diagnosis and sensory sensitivity with course failure, were consistent with the expectation that the associations would be stronger for women than for men.

## Discussion

This study was concerned with the role of autistic traits in academic and social outcomes for college students. Specifically, we were interested in whether autistic traits predict outcomes independent of the diagnosis, whether traits are more strongly associated with outcomes for students who have not received an autism diagnosis, and whether the associations vary by gender.

Our findings confirm prior evidence for the association of autistic traits with social outcomes (e.g., Jobe & White 2007; Wainer et al., 2013) and extend that evidence to academic outcomes. This extension is important inasmuch as academic outcomes such as GPA, course failure, and academic difficulties strongly predict college graduation (DeBerard et al., 2004). Although we could not measure graduation directly in this cross-sectional study, our results suggest that autistic traits, rather than autism diagnosis alone, are important determinants of degree attainment.

When we disaggregated results by trait subscales, we expected mentalizing deficits to be more closely associated with academic outcomes than social anxiety and sensory reactivity, and mentalizing deficits and social anxiety to be more closely associated with social outcomes than sensory reactivity. Our results were only partially consistent with these expectations. Consistent with what we expected, of the subscales, only mentalizing deficits was associated with GPA in the total sample. However, all subscale scores were significantly associated with course failure and academic difficulties. Moreover, all subscale scores—even sensory reactivity—were also significantly associated with all social outcomes.

One possible explanation for the consistent associations across subscales is methodological. The correlations among the subscale scores were relatively high—ranging from 0.45 to 0.59—which suggests empirical overlap among the trait dimensions that they measure. It is also possible that our expectations were simply wrong: that each of the major dimensions of traits plays a role in the academic and social disadvantages that autistic college students experience. For example, anxiety has been associated with college GPA and dropout in general (Eisenberg et al., 2009) and with poorer college adjustment among autistic students (Lei et al., 2020). Sensory sensitivities may disrupt social relationships to the extent that they restrict the range of activities in which autistic students can comfortably engage (Thye et al., 2018). In short, specific types of traits and outcomes do not appear to be as tightly coupled as we expected.

Our results also were not consistent with our expectation that autistic traits would be more strongly associated with academic outcomes for students who had not received a diagnosis than for those who had. Although the associations of autistic traits were more often significant among students without a diagnosis, the associations were not significantly different based on diagnostic status and, for course failure and academic difficulties, the coefficients were often *larger* for students with a diagnosis. This suggests that, even among the more homogeneous group of students who have received a diagnosis, variation in autistic traits contributes to variation in academic outcomes.

The results were less straightforward for social outcomes. The directions of the associations of autistic traits were more consistent with expectation for students without an autism diagnosis than for those with a diagnosis. For example, autistic traits were associated with higher odds of having a confidant for students with a diagnosis and lower odds of having a confidant for students without a diagnosis. Autistic traits were also associated with higher odds of social exclusion only for students without a diagnosis. Tempering our conclusions is the finding that most of the coefficient differences for social outcomes were not statistically significant. Only the coefficients for predicting the presence of a confidant were significantly different and, in that case, because of the surprising finding among students with a diagnosis.

Our analysis could not address the question of why autistic traits increase the odds of having a confidant among students with a diagnosis. One possibility is that students with an autism diagnosis have access to social programs, support groups, or individual therapeutic interventions that facilitate the development of close relationships. Some, although not all, of the institutions that participated in this study offer support programs specific to autism. Another possibility is that the result is a function of the specific measure we used. The question we asked (“Is there anyone you can really open up to about your most private feelings without having to hold them back?”) is commonly used in major social surveys to assess availability of a close confidant. Based on evidence that the availability of an intimate, confiding relationship is one of the most powerful measures of support (Thoits, 1995), this measure is a key indicator of the availability of social support. Despite the general effectiveness of this measure, it is possible that the types of support it measures are different for autistic and neurotypical students. If, for example, the confidants for autistic students are more likely to be family, faculty, or staff (Cai & Richdale, 2016) whereas those for neurotypical students are more likely to be peers, the higher probability of reporting a confidant for autistic students could reflect support seeking rather than support receipt.

We also considered whether the associations differ by gender. One set of studies led us to expect that autistic traits would be more strongly associated with social outcomes for young women than for young men, based on the argument that autistic traits are less compatible with friendship expectations for women than for men (Felmlee et al., 2012; Hall, 2011; Koenig & Tsatsanis, 2005). Contrary to this expectation, none of the coefficients for social outcomes was significantly different by gender. Indeed, although the associations of an autism diagnosis and autistic traits with academic outcomes were much more often significant for women than men, the only significant gender differences were for course failure.

The significant associations of the autism indicators with academic outcomes for women may reflect the greater presence of comorbid conditions in that group. Among entering college freshmen with autism, young women report higher levels of feeling overwhelmed and depressed than young men and are more likely to report a mental health disorder (Kreiser & White, 2015; Sturm & Kasari 2019). Anxiety and depression are associated with poor academic performance in college (Eisenberg et al., 2009). To the extent that autistic women college students experience higher prevalence of those disorders, we would expect them to struggle more academically than their male counterparts.

The absence of gender differences in the associations with social outcomes aligns with prior studies that find that gender, more than autism, shapes the friendship experiences of autistic boys and girls in adolescence (Sedgewick et al., 2019). Although Sedgewick and colleagues found that there are gender differences in the friendship experiences of autistic boys and girls, many of the friendship challenges that autistic, adolescent girls report (e.g., relational conflict) were also reported by neurotypical girls. Our results support the implied conclusion of that study: that there are differences in friendships based on gender and on autism but that gender and autism status do not interact when predicting friendships.

Our study has several strengths, including a relatively large sample of students with autism diagnoses, a comparison sample of students without a diagnosis, and nationally validated measures of academic and social outcomes. At the same time, our study has several limitations that warrant mention. First, as noted, although we rely on measures that have been validated in national, general population samples, the measures were not developed specifically for autistic students and, in some cases, may be less valid for that group. Second, and more generally, our reliance on self-report measures leaves us open to the criticism that any observed associations may reflect differences in reporting tendencies rather than true associations. Third, our measure of autistic traits—although validated in previous studies—may fail to capture the traits that matter most for college outcomes. For example, it does not measure pragmatic language difficulties, which have been associated with college student outcomes in prior research (Trevisan & Birmingham, 2015). Fourth and finally, our study was conducted in a specific set of postsecondary institutions in single state and may not be representative of the experiences of students at other institutions and in other states.

These limitations aside, our results demonstrate that autistic traits affect academic and social experiences of college students independent of an autism diagnosis, and that autistic traits are associated with more negative social outcomes for students without a diagnosis. These results support the development of both academic and non-academic programs and services (Accardo et al., 2019) for students with autistic traits regardless of diagnostic status (see also VanBergeijk et al., 2008). These might include programs to educate instructors and residence hall staff about autistic traits as well as academic and social support programs that are available to a broader range of students than disability services offices typically serve (Gobbo & Shmulsky, 2014; Zeedyk et al., 2019).

To be successful, such programs will have to reach students who have not received a diagnosis and, therefore, cannot depend on the formal disability accommodation application processes that many institutions currently require (Viezel et al., 2020). This, in turn, may require that institutions devote additional resources to identifying students in need of assistance whose autistic traits have not been formally diagnosed (Reed et al., 2016). Beyond enhanced identification, some approaches institutions might consider include regular assessments of the quality of students’ available social support, programs to strengthen peer communities, and outreach to student organizations and residential communities (Hefner & Eisenberg, 2009). These kinds of approaches are consistent with movements within higher education to adopt more systemic, environmental approaches to student well-being (Travia et al., 2020) and would have the benefit of serving students with social support deficits who might not otherwise come to the notice of institutional authorities.

## Footnotes

1 At public institutions, only about 60% of undergraduate students are 24 years of age or younger. Private institutions skew to younger ages. Thus, at public institutions, younger than 35 is considered “typical” college age.
