# Correction: Autistic Traits and College Adjustment

**DOI:** 10.1007/s10803-025-06792-1

**Published:** 2025-03-26

**Authors:** Jane D. McLeod, Elizabeth M. Anderson

**Affiliations:** Department of Sociology, Indiana University, Bloomington, IN USA

**Correction to: Journal of Autism and Developmental Disorders (2023) 53:3475–3492** 10.1007/s10803-022-05632-w

In this article, errors have occurred in Tables 7 and 8, and in the section heading “Do the associations differ by gender?” of the “Results” section.

Both the incorrect and corrected information are stated below.

The Students who reported “other” gender identities were mistakenly included with men in the gender-specific analyses. As a result, the significance of two coefficients have changed in the Tables 7 and 8: (1) Mentalizing deficits are now associated with a higher likelihood of course failure for men; (2) The autism diagnosis is no longer significantly associated with social exclusion for men.

Incorrect Table 7:

**Table 7:** Coefficients from regressions of academic outcomes on autism indicators for women and men

	Panel C: Women (N = 1,703)					Panel D: Men (N = 1,026)				
**GPA**	**M1**	**M2**	**M3**	**M4**	**M5**	**M1**	**M2**	**M3**	**M4**	**M5**
Autism diagnosis	-0.23 (0.16)	-0.14 (0.14)	-0.15 (0.15)	-0.22 (0.15)	-0.17 (0.14)	-0.09 (0.07)	-0.07 (0.08)	-0.06 (0.08)	-0.08 (0.07)	-0.09 (0.08)
Autistic traits	–	-0.04^*^ (0.02)	–	–	–	–	-0.02 (0.01)	–	–	–
Mentalizing deficits	–	–	-0.05^**^ (0.02)	–	–	–	–	-0.02 (0.01)	–	–
Social anxiety	–	–	–	-0.01 (0.02)	–	–	–	–	-0.01 (0.02)	–
Sensory reactivity	–	–	–	–	-0.04^*^ (0.02)	–	–	–	–	0.00 (0.02)
**Failed a course**	**M1**	**M2**	**M3**	**M4**	**M5**	**M1**	**M2**	**M3**	**M4**	**M5**
Autism diagnosis	2.62^***^ (0.67)	1.84^*^ (0.51)	1.96^**^ (0.51)	2.35^**^ (0.62)	2.18^**^ (0.62)	0.83 (0.24)	0.71 (0.19)	0.69 (0.18)	0.75 (0.21)	0.83 (0.23)
Autistic traits	–	1.19^***^ (0.05)	–	–	–	–	1.12 (0.08)	–	–	–
Mentalizing deficits	–	–	1.20^***^ (0.05)	–	–	–	–	1.14 (0.09)	–	–
Social anxiety	–	–	–	1.08 (0.05)	–	–	–	–	1.12 (0.08)	–
Sensory reactivity	–	–	–	–	1.13^**^ (0.05)	–	–	–	–	1.00 (0.04)
**Academic difficulties**	**M1**	**M2**	**M3**	**M4**	**M5**	**M1**	**M2**	**M3**	**M4**	**M5**
Autism diagnosis	0.22^*^ (0.07)	-0.16 (0.07)	-0.12 (0.09)	0.03 (0.08)	0.05 (0.08)	0.13 (0.08)	-0.11 (0.08)	-0.08 (0.08)	0.00 (0.08)	-0.01 (0.09)
Autistic traits	–	0.18^***^ (0.01)	–	–	–	–	0.19^***^ (0.03)	–	–	–
Mentalizing deficits	–	–	0.17^***^ (0.02)	–	–	–	–	0.16^***^ (0.02)	–	–
Social anxiety	–	–	–	0.14^***^ (0.01)	–	–	–	–	0.16^***^ (0.02)	–
Sensory reactivity	–	–	–	–	0.11^***^ (0.01)	–	–	–	–	0.13^**^ (0.03)

Notes: Coefficients for GPA and academic difficulties are from OLS regressions. Coefficients for course failure are exponentiated, from logistic regressions. Sample sizes vary slightly from missingness in the dependent variables and autism scales

Corrected Table 7:

**Table 7:** Coefficients from regressions of academic outcomes on autism indicators for women and men

	Panel C: Women (N = 1,703)					Panel D: Men (N = 994)				
**GPA**	**M1**	**M2**	**M3**	**M4**	**M5**	**M1**	**M2**	**M3**	**M4**	**M5**
Autism diagnosis	− 0.23 (0.16)	− 0.14 (0.14)	− 0.15 (0.15)	− 0.22 (0.15)	− 0.17 (0.14)	− 0.09 (0.08)	− 0.06 (0.10)	− 0.05 (0.10)	− 0.08 (0.08)	− 0.09 (0.09)
Autistic traits	–	− 0.04^*^ (0.02)	–	–	–	–	− 0.02 (0.02)	–	–	–
Mentalizing deficits	–	–	− 0.05^**^ (0.02)	–	–	–	–	− 0.02 (0.02)	–	–
Social anxiety	–	–	–	− 0.01 (0.02)	–	–	–	–	− 0.01 (0.02)	–
Sensory reactivity	–	–	–	–	− 0.04^*^ (0.02)	–	–	–	–	0.00 (0.02)
**Failed a course**	**M1**	**M2**	**M3**	**M4**	**M5**	**M1**	**M2**	**M3**	**M4**	**M5**
Autism diagnosis	2.62^***^ (0.67)	1.84^*^ (0.51)	1.96^**^ (0.51)	2.35^**^ (0.62)	2.18^**^ (0.62)	0.74 (0.30)	0.63 (0.25)	0.61 (0.23)	0.67 (0.27)	0.76 (0.30)
Autistic traits	–	1.19^***^ (0.05)	–	–	–	–	1.12 (0.07)	–	–	–
Mentalizing deficits	–	–	1.20^***^ (0.05)	–	–	–	–	1.15* (0.07)	–	–
Social anxiety	–	–	–	1.08 (0.05)	–	–	–	–	1.12 (0.08)	–
Sensory reactivity	–	–	–	–	1.13^**^ (0.05)	–	–	–	–	0.97 (0.03)
**Academic difficulties**	**M1**	**M2**	**M3**	**M4**	**M5**	**M1**	**M2**	**M3**	**M4**	**M5**
Autism diagnosis	0.22^*^ (0.07)	− 0.16 (0.07)	− 0.12 (0.09)	0.03 (0.08)	0.05 (0.08)	0.08 (0.09)	− 0.16 (0.09)	− 0.13 (0.09)	− 0.05 (0.08)	− 0.05 (0.10)
Autistic traits	–	0.18^***^ (0.01)	–	–	–	–	0.18^***^ (0.02)	–	–	–
Mentalizing deficits	–	–	0.17^***^ (0.02)	–	–	–	–	0.15^***^ (0.02)	–	–
Social anxiety	–	–	–	0.14^***^ (0.01)	–	–	–	–	0.15^***^ (0.01)	–
Sensory reactivity	–	–	–	–	0.11^***^ (0.01)	–	–	–	–	0.12^**^ (0.03)

Notes: Coefficients for GPA and academic difficulties are from OLS regressions. Coefficients for course failure are exponentiated, from logistic regressions. Sample sizes vary slightly from missingness in the dependent variables and autism scales

Incorrect Table 8:

**Table 8:** Coefficients from regressions of social outcomes on autism indicators for women and men

	Panel C: Women (N = 1,703)					Panel D: Men (N = 1,026)				
**Confidant**	**M1**	**M2**	**M3**	**M4**	**M5**	**M1**	**M2**	**M3**	**M4**	**M5**
Autism diagnosis	0.56 (0.29)	1.25 (0.61)	1.04 (0.56)	0.96 (0.43)	0.78 (0.42)	0.55 (0.23)	0.83 (0.42)	0.77 (0.39)	0.78 (0.38)	0.57 (0.26)
Autistic traits	–	0.67^***^ (0.06)	–	–	–	–	0.73^***^ (0.07)	–	–	–
Mentalizing deficits	–	–	0.72^***^ (0.07)	–	–	–	–	0.78^**^ (0.06)	–	–
Social anxiety	–	–	–	0.66^***^ (0.03)	–	–	–	–	0.63^***^ (0.08)	–
Sensory reactivity	–	–	–	–	0.81^*^ (0.07)	–	–	–	–	0.98 (0.07)
**Friendship Quality**	**M1**	**M2**	**M3**	**M4**	**M5**	**M1**	**M2**	**M3**	**M4**	**M5**
Autism diagnosis	-0.18 (0.13)	0.18 (0.11)	0.09 (0.10)	0.12 (0.11)	-0.04 (0.14)	-0.11 (0.09)	0.12 (0.07)	0.03 (0.09)	0.09 (0.06)	-0.02 (0.08)
Autistic traits	–	-0.17^***^ (0.01)	–	–	–	–	-0.17^***^ (0.02)	–	–	–
Mentalizing deficits	–	–	-0.11^***^ (0.01)	–	–	–	–	0.10^***^ (0.01)	–	–
Social anxiety	–	–	–	-0.21^***^ (0.02)	–	–	–	–	-0.24^***^ (0.03)	–
Sensory reactivity	–	–	–	–	-0.09^***^ (0.01)	–	–	–	–	-0.08^**^ (0.02)
**Social Exclusion**	**M1**	**M2**	**M3**	**M4**	**M5**	**M1**	**M2**	**M3**	**M4**	**M5**
Autism diagnosis	4.53^*^ (3.20)	2.53 (1.93)	2.67 (2.04)	3.97 (3.03)	3.01 (2.18)	2.38^**^ (0.79)	1.24 (0.54)	1.25 (0.55)	1.85 (0.70)	1.54 (0.59)
Autistic traits	–	1.32^***^ (0.12)	–	–	–	–	1.70^***^ (0.19)	–	–	–
Mentalizing deficits	–	–	1.34^***^ (0.11)	–	–	–	–	1.70^***^ (0.16)	–	–
Social anxiety	–	–	–	1.10 (0.09)	–	–	–	–	1.41^**^ (0.17)	–
Sensory reactivity	–	–	–	–	1.27^**^ (0.11)	–	–	–	–	1.44^***^ (0.14)

Notes: Coefficients for friendship quality are from OLS regressions. Coefficients for confidant and social exclusion are exponentiated, from logistic regressions. Sample sizes vary slightly from missingness in the dependent variables and autism scales

Corrected Table 8:

**Table 8:** Coefficients from regressions of social outcomes on autism indicators for women and men

	Panel C: Women (N = 1,703)					Panel D: Men (N = 994)				
**Confidant**	**M1**	**M2**	**M3**	**M4**	**M5**	**M1**	**M2**	**M3**	**M4**	**M5**
Autism diagnosis	0.56 (0.29)	1.25 (0.61)	1.04 (0.56)	0.96 (0.43)	0.78 (0.42)	0.53 (0.23)	0.83 (0.42)	0.78 (0.39)	0.77 (0.38)	0.55 (0.26)
Autistic traits	–	0.67^***^ (0.06)	–	–	–	–	0.72^***^ (0.07)	–	–	–
Mentalizing deficits	–	–	0.72^***^ (0.07)	–	–	–	–	0.76^**^ (0.06)	–	–
Social anxiety	–	–	–	0.66^***^ (0.03)	–	–	–	–	0.64^***^ (0.08)	–
Sensory reactivity	–	–	–	–	0.81^*^ (0.07)	–	–	–	–	0.98 (0.08)
**Friendship Quality**	**M1**	**M2**	**M3**	**M4**	**M5**	**M1**	**M2**	**M3**	**M4**	**M5**
Autism diagnosis	− 0.18 (0.13)	0.18 (0.11)	0.09 (0.10)	0.12 (0.11)	− 0.04 (0.14)	− 0.12 (0.10)	0.10 (0.08)	0.01 (0.09)	0.09 (0.07)	− 0.03 (0.09)
Autistic traits	–	− 0.17^***^ (0.01)	–	–	–	–	− 0.16^***^ (0.02)	–	–	–
Mentalizing deficits	–	–	− 0.11^***^ (0.01)	–	–	–	–	− 0.09^***^ (0.01)	–	–
Social anxiety	–	–	–	− 0.21^***^ (0.02)	–	–	–	–	− 0.23^***^ (0.03)	–
Sensory reactivity	–	–	–	–	− 0.09^***^ (0.01)	–	–	–	–	− 0.07^**^ (0.02)
**Social Exclusion**	**M1**	**M2**	**M3**	**M4**	**M5**	**M1**	**M2**	**M3**	**M4**	**M5**
Autism diagnosis	4.53^*^ (3.20)	2.53 (1.93)	2.67 (2.04)	3.97 (3.03)	3.01 (2.18)	1.45 (0.70)	0.71 (0.38)	0.72 (0.38)	1.05 (0.56)	0.90 (0.47)
Autistic traits	–	1.32^***^ (0.12)	–	–	–	–	1.72^***^ (0.19)	–	–	–
Mentalizing deficits	–	–	1.34^***^ (0.11)	–	–	–	–	1.73^***^ (0.16)	–	–
Social anxiety	–	–	–	1.10 (0.09)	–	–	–	–	1.44^**^ (0.16)	–
Sensory reactivity	–	–	–	–	1.27^**^ (0.11)	–	–	–	–	1.43^***^ (0.15)

Notes: Coefficients for friendship quality are from OLS regressions. Coefficients for confidant and social exclusion are exponentiated, from logistic regressions. Sample sizes vary slightly from missingness in the dependent variables and autism scales

In the “Results” section, under the heading “Do the associations differ by gender?” in the second paragraph, the sentence “In contrast, for men, the total score and all subscale scores were only associated with higher levels of academic difficulties” should read as “In contrast, for men, the total score and all subscale scores were associated with higher levels of academic difficulties and only mentalizing deficits were associated with the likelihood of course failure”.

In the “Results” section, under the heading “Do the associations differ by gender?” the third paragraph was incorrectly written as “The pattern of associations for social outcomes was more similar for women and men. The autism diagnosis was significantly associated with social exclusion for both women and men. Although the odds-ratio was larger for women than for men, the difference was not significant, probably because of the relatively small number of young women with an autism diagnosis. In addition, the total trait score and most subscale scores were associated with a lower likelihood of having a confidant, lower friendship quality, and a higher likelihood of being socially excluded for both women and men. Despite these differences in the within-group results, none of the coefficients was significantly different by gender.” but should read as “The pattern of associations for social outcomes was more similar for women and men. The total trait score and most subscale scores were associated with a lower likelihood of having a confidant, lower friendship quality, and a higher likelihood of being socially excluded for both women and men. The autism diagnosis was significantly associated with social exclusion only for women. Despite this difference in the within-group results, none of the coefficients was significantly different by gender.”

